# Supplementary material for: Assessment of PRMT6-dependent alternative splicing in pluripotent and differentiating NT2/D1 cells
Source: Life Sci Alliance. 2025 Feb 3;8(4):e202402946. doi: 10.26508/lsa.202402946 (PMC11791029; doi:10.26508/lsa.202402946)
Supplement: Supplementary file 1 [file LSA-2024-02946_SdataF1.pptx]

## Slide 1
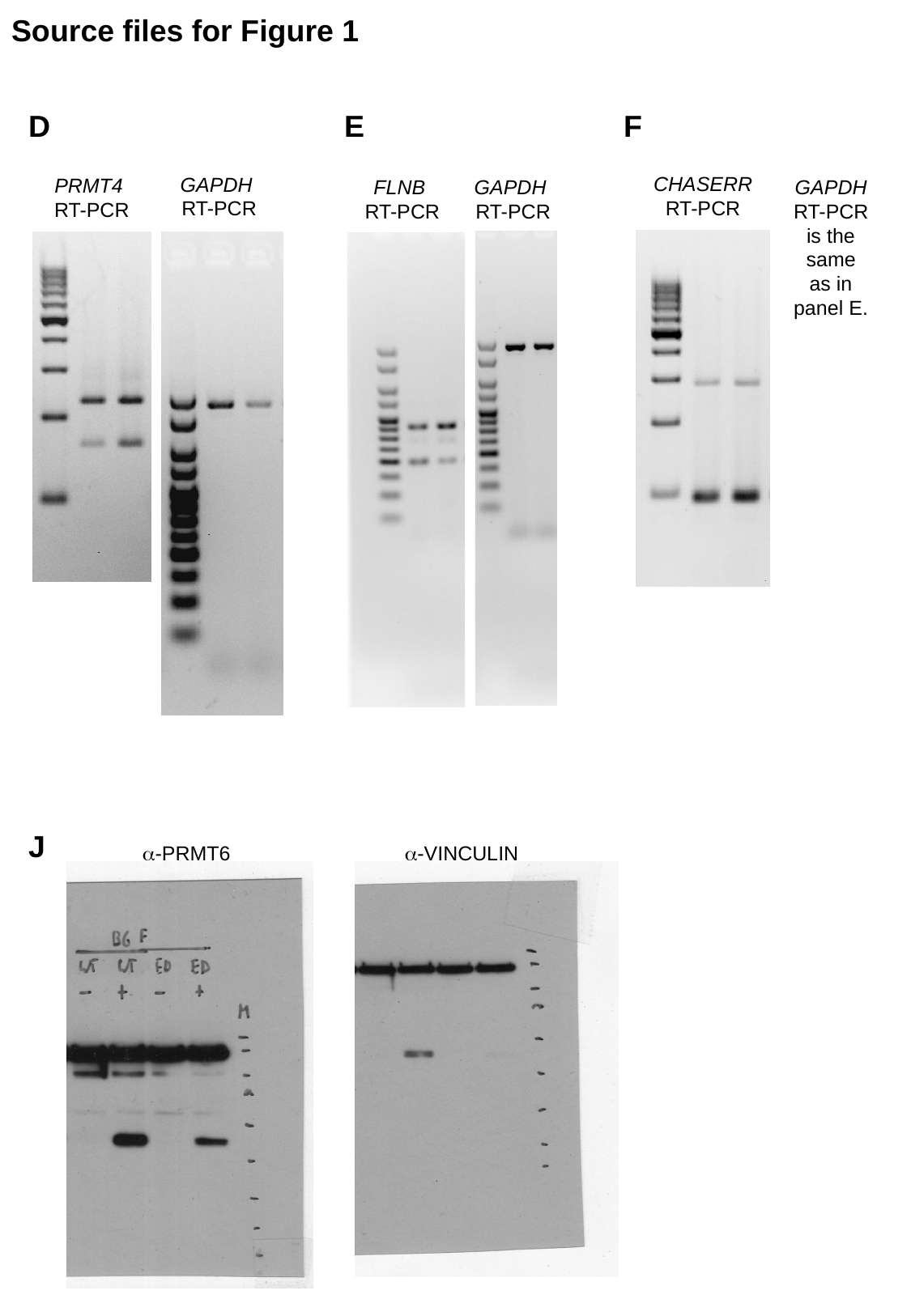

Source files for Figure 1
D
E
F
CHASERR
RT-PCR
GAPDH
RT-PCR
PRMT4
RT-PCR
FLNB
RT-PCR
GAPDH
RT-PCR
GAPDH
RT-PCR
is the same as in panel E.
J
a-PRMT6
a-VINCULIN
