# Supplementary figures and images for: Assessment of PRMT6-dependent alternative splicing in pluripotent and differentiating NT2/D1 cells
Source: Life Sci Alliance. 2025 Feb 3;8(4):e202402946. doi: 10.26508/lsa.202402946 (PMC11791029; doi:10.26508/lsa.202402946)

## Slide 1
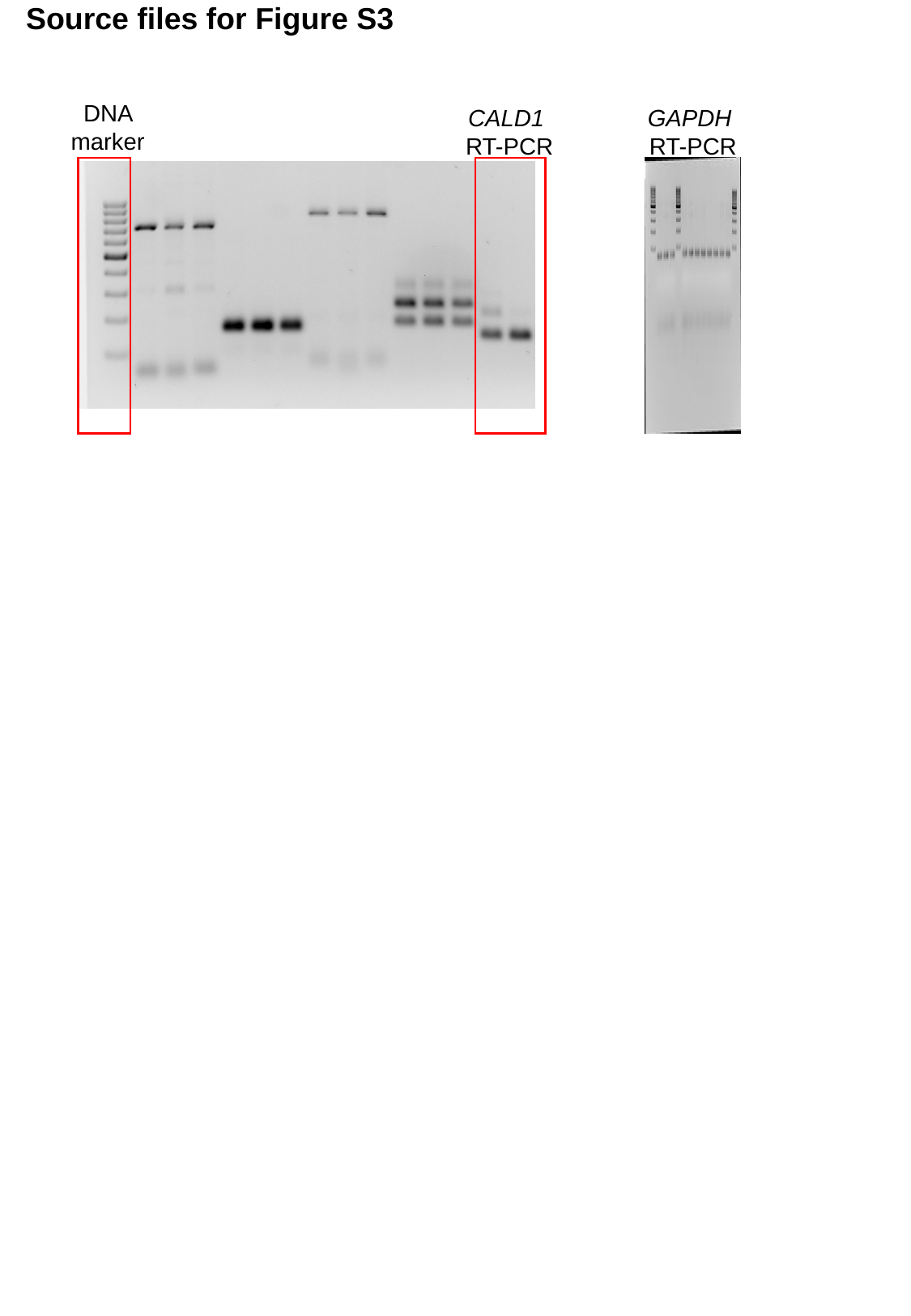

Source files for Figure S3
DNA marker
CALD1
RT-PCR
GAPDH
RT-PCR

Supplement: Supplementary file 3 [file LSA-2024-02946_SdataFS3.pptx]

## Slide 1
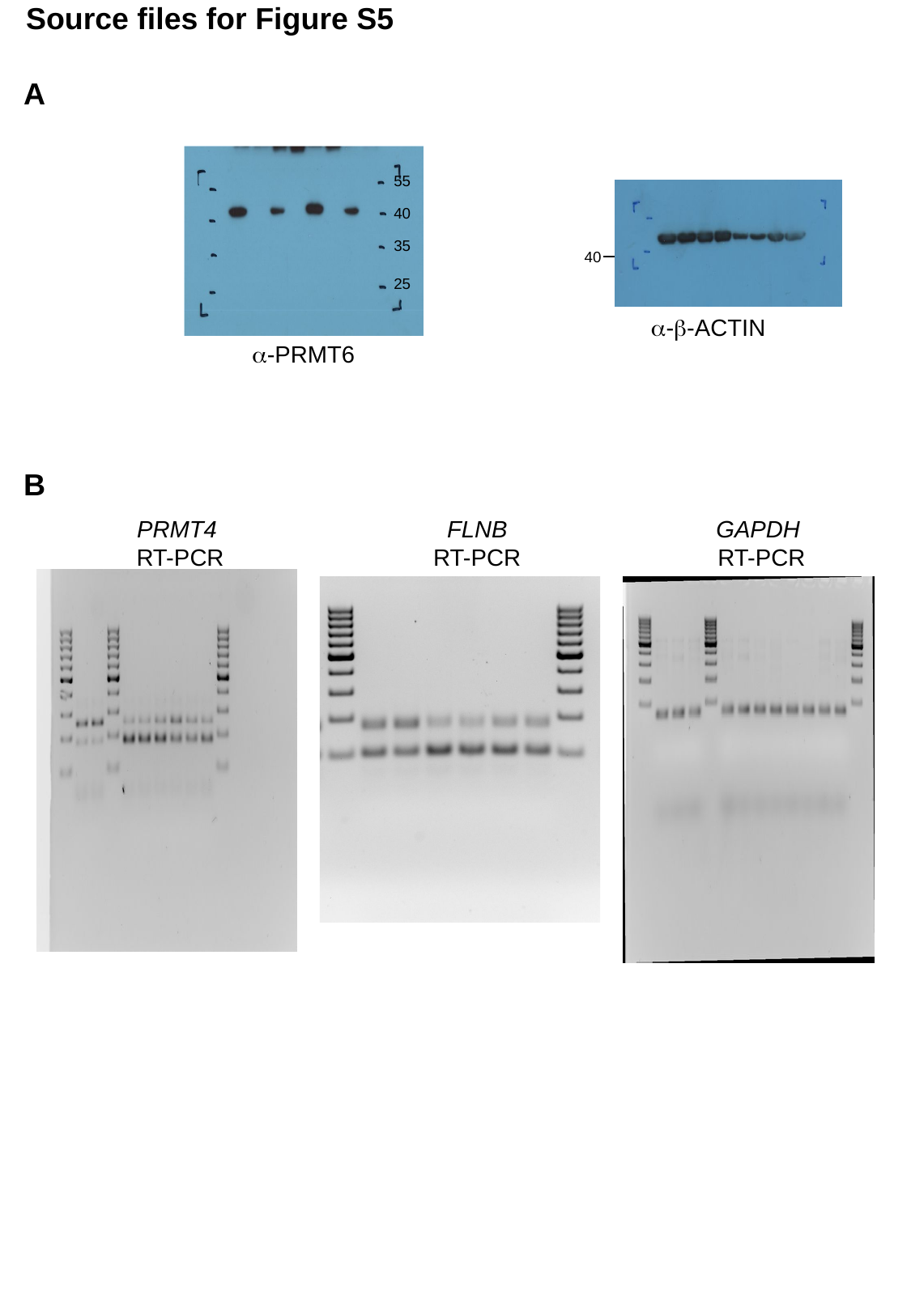

Source files for Figure S5
A
55
40
35
40
25
a-b-ACTIN
a-PRMT6
B
PRMT4
RT-PCR
FLNB
RT-PCR
GAPDH
RT-PCR

Supplement: Supplementary file 4 [file LSA-2024-02946_SdataFS5.pptx]

## Slide 1
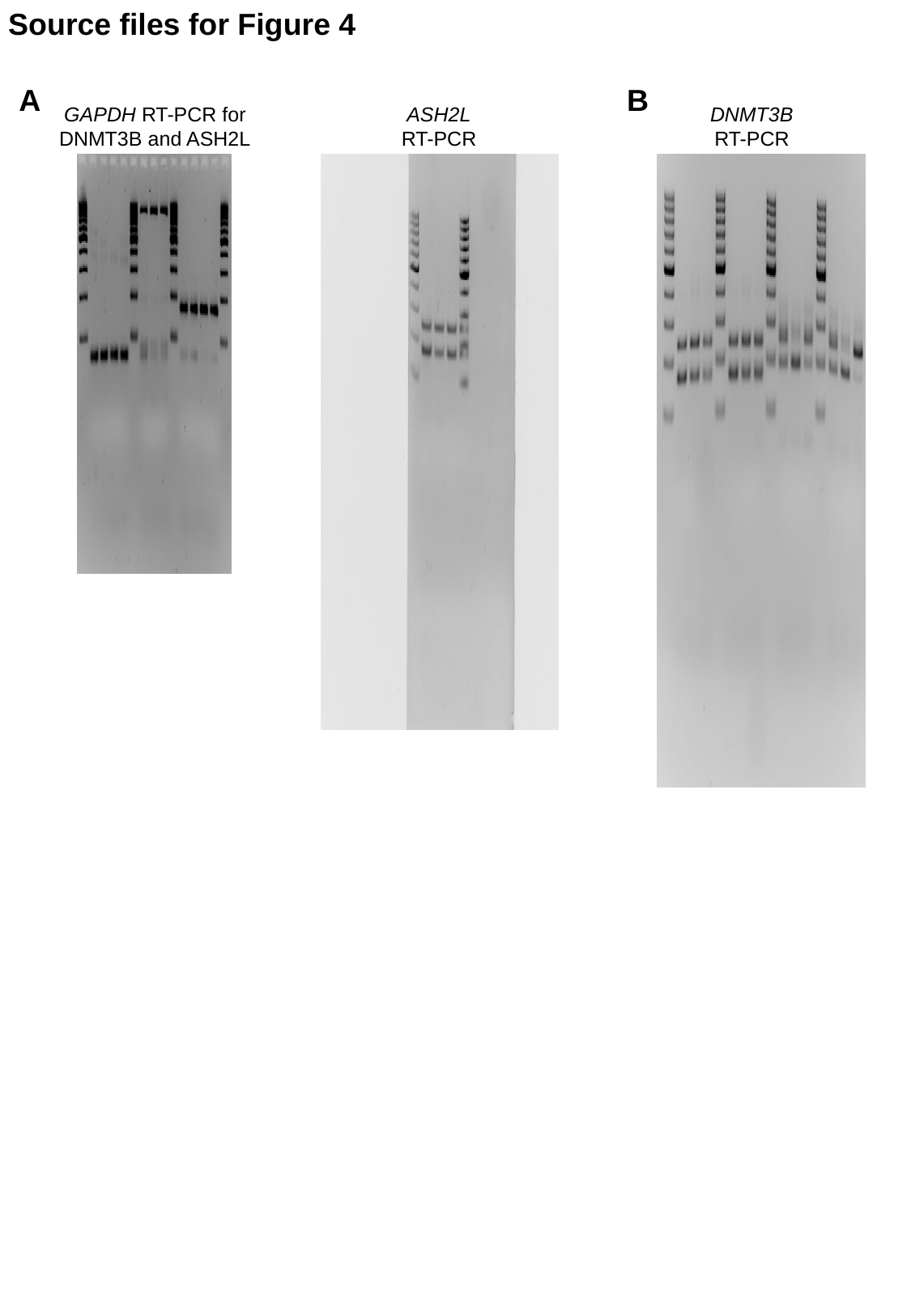

Source files for Figure 4
A
B
GAPDH RT-PCR for
DNMT3B and ASH2L
ASH2L
RT-PCR
DNMT3B
RT-PCR

Supplement: Supplementary file 5 [file LSA-2024-02946_SdataF4.pptx]
