# Supplementary material for: Assessment of PRMT6-dependent alternative splicing in pluripotent and differentiating NT2/D1 cells
Source: Life Sci Alliance. 2025 Feb 3;8(4):e202402946. doi: 10.26508/lsa.202402946 (PMC11791029; doi:10.26508/lsa.202402946)
Supplement: Supplementary file 6 [file LSA-2024-02946_SdataFS11.pptx]

## Slide 1
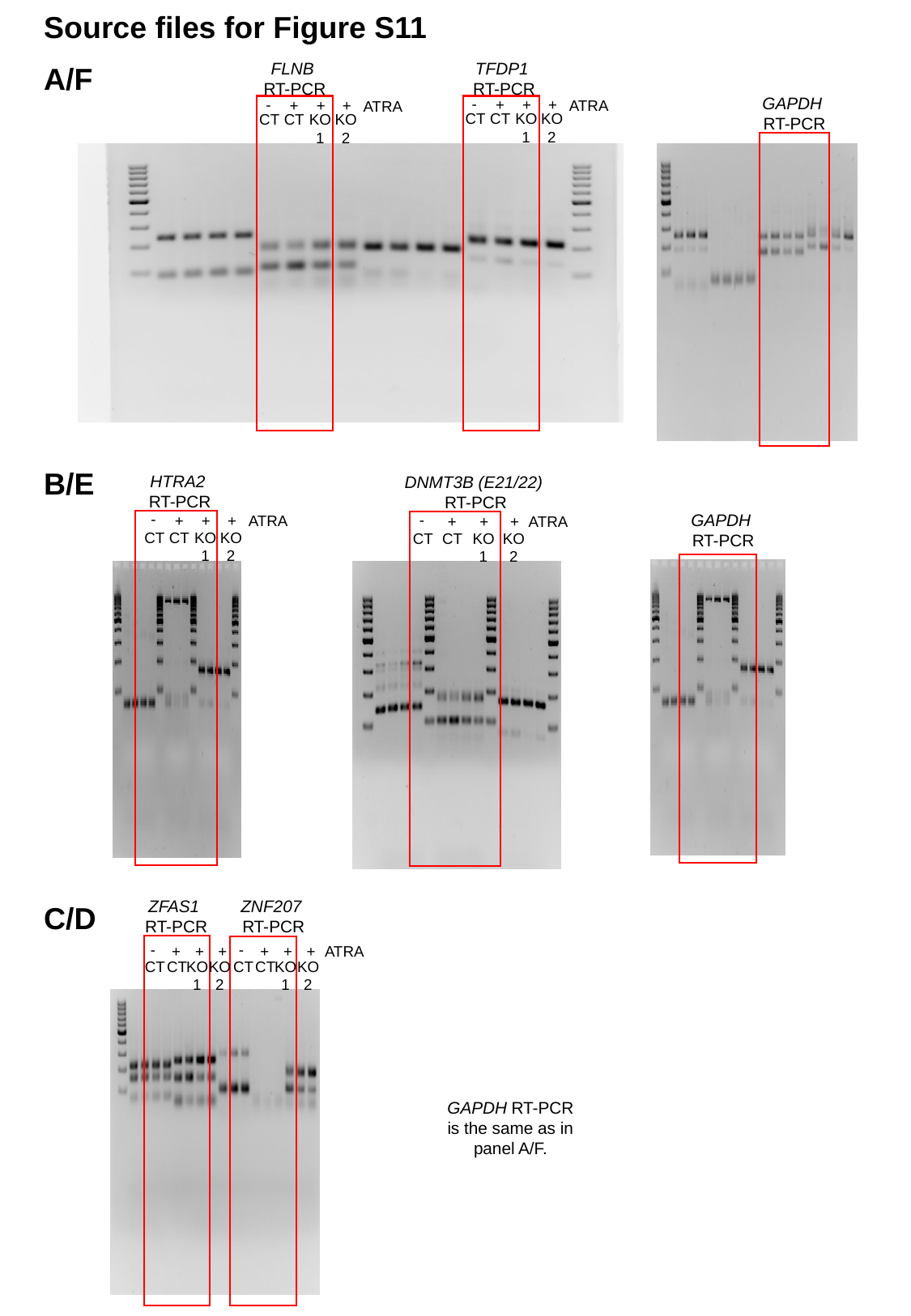

Source files for Figure S11
FLNB
RT-PCR
TFDP1
RT-PCR
GAPDH
RT-PCR
-
+
+
+
ATRA
CT
CT
KO
1
KO
2
-
+
+
+
ATRA
CT
CT
KO
1
KO
2
A/F
B/E
HTRA2
RT-PCR
DNMT3B (E21/22)
RT-PCR
-
GAPDH
RT-PCR
-
+
+
+
ATRA
+
+
+
ATRA
KO
1
KO
2
CT
CT
KO
1
KO
2
CT
CT
ZFAS1
RT-PCR
ZNF207
RT-PCR
C/D
-
-
+
+
+
+
+
+
ATRA
KO
1
KO
2
KO
1
KO
2
CT
CT
CT
CT
GAPDH RT-PCR
is the same as in panel A/F.
